# Analysis and comparison of 2D fingerprints: insights into database screening performance using eight fingerprint methods

**DOI:** 10.1186/1758-2946-3-S1-P1

**Published:** 2011-04-19

**Authors:** J Duan, M Sastry, SL Dixon, JF Lowrie, W Sherman

**Affiliations:** 1 Schrödinger GmbH, Dynamostr. 13, Mannheim, 68165, Germany; 2 Schrödinger Inc. 120 West 45th Street, 17th Floor, New York, NY 10036-4041, USA

## 

Virtual screening is a widely used strategy in modern drug discovery and 2D fingerprint similarity is an important tool that has been successfully applied to retrieve active compounds from large datasets. However, it is not always straightforward to select appropriate fingerprint method and associated settings for a given problem. Here, we applied eight different fingerprint methods, as implemented in the new cheminformatics package Canvas, on a well validated dataset covering five targets. The fingerprint methods include Linear, Dendritic, Radial, MACCS, MOLPRINT2D, Pairwise, Triplet, and Torsion. We find that most fingerprints have similar retrieval rates on average; however, each has special characteristics that distinguish its performance on different query molecules and ligand sets. For example, some fingerprints exhibit a significant ligand size dependency whereas others are more robust with respect to variations in the query or active compounds. In cases where little information is known about the active ligands, MOLPRINT2D fingerprints produce the highest average retrieval actives. When multiple queries are available, we find that a fingerprint averaged over all query molecules is generally superior to fingerprints derived from single queries. Finally, a complementarity metric is proposed to determine which fingerprint methods can be combined to improve screening results.

A more systematic virtual screening study has also been conducted to investigate the interrelation between eight fingerprinting methods, eleven atomtyping schemes, seven bit scaling rules, and four similarity metrics. In total, 24,068 virtual screens were performed to assess the effectiveness of each combination of options to identify active ligands in a database screen performed on 11 pharmaceutically relevant targets. Significant variations in enrichments were observed with all explored parameters. In general, fingerprints such as MOLPRINT2D and Dendritic that contain information about local environment beyond simple linear paths outperformed other fingerprint methods. Atomtyping schemes with more specific information were generally superior to more generic atomtyping schemes. With the best identified settings, enrichment factors across all targets could be improved considerably. No single combination of settings performed optimally on all targets and therefore we provide recommendations to improve enrichments based on different requirements.

